# Supplementary material for: Coevolutionary constraints of Zika virus nonstructural protein 5 replication and interferon antagonism activities
Source: bioRxiv. 2025 Dec 23:2025.12.23.696258. Preprint. [Version 1] doi: 10.64898/2025.12.23.696258 (PMC12776046; doi:10.64898/2025.12.23.696258)

bioRxiv preprint doi: https://doi.org/10.64898/2025.12.23.696258; this version posted December 24, 2025. The copyright holder for this preprint (which was not certified by peer review) is the author/funder, who has granted bioRxiv a license to display the preprint in perpetuity. It is made available under aCC-BY 4.0 International license.

Supplementary Fig. 1: Structural model of the ZIKV NS5–STAT2 complex. Composite structural model showing the interaction between ZIKV NS5 and human STAT2. NS5 is displayed as a space-filling model with the methyltransferase (light gray) and RNA-dependent RNA polymerase (dark gray) domains indicated. STAT2 is shown as a ribbon diagram, with the N-terminal domain (ND) in light blue and the coiled-coil domain (CCD) in marine blue. NS5 residues that lie in close proximity defined to STAT2 are highlighted in yellow. Two orientations of the complex are shown, rotated 180° around the vertical axis to reveal both sides of the interface. Structures were modeled using available cryo-EM and crystallographic data (PDB: 6WCZ, 6UX2).

Supplementary Fig. 2: Impacts of single amino acid mutations on ZIKV NS5 IFN antagonism. Each panel shows the relative IFN signaling in STAT2 knockout 293T cells in the presence of single amino acid substitutions at the residue indicated on the x-axis. Each panel includes the controls: wild type NS5, the antagonism deficient D734R mutant, an equal mixture of wild type and D734R, 'no NS5', and 'no NS5 or STAT2'. All transfections except the 'no NS5 or

bioRxiv preprint doi: https://doi.org/10.64898/2025.12.23.696258; this version posted December 24, 2025. The copyright holder for this preprint (which was not certified by peer review) is the author/funder, who has granted bioRxiv a license to display the preprint in perpetuity. It is made available under aCC-BY 4.0 International license.

STAT2' condition include human STAT2 to complement the KO. Asterisks indicate statistical significance determined by multiple unpaired t-tests with Holm-Šidák method to correct for multiple comparisons.

Supplementary Fig. 3: Quality control and validation of ZIKV NS5 deep mutational scanning libraries. The NS5 coding region was divided into eight consecutive 'tiles' for DMS. Tile numbers and amino-acid ranges are labeled to the left of each row, with the remaining panels in each row showing: **(a)** Per-codon mutation frequencies measured in plasmid DNA (input) and after viral selection are shown, with mutation types indicated (nonsynonymous = black, synonymous = blue, stop = orange). **(b)** Cumulative count distributions for amino acid substitutions (black) and codons (orange) are displayed for plasmid libraries and post-selection viral samples. **(c)** Scatterplots show pairwise correlations of codon frequencies between independently generated plasmid libraries, with Pearson correlation coefficients (R) indicated.

bioRxiv preprint doi: https://doi.org/10.64898/2025.12.23.696258; this version posted December 24, 2025. The copyright holder for this preprint (which was not certified by peer review) is the author/funder, who has granted bioRxiv a license to display the preprint in perpetuity. It is made available under aCC-BY 4.0 International license.

Supplementary Figure 1

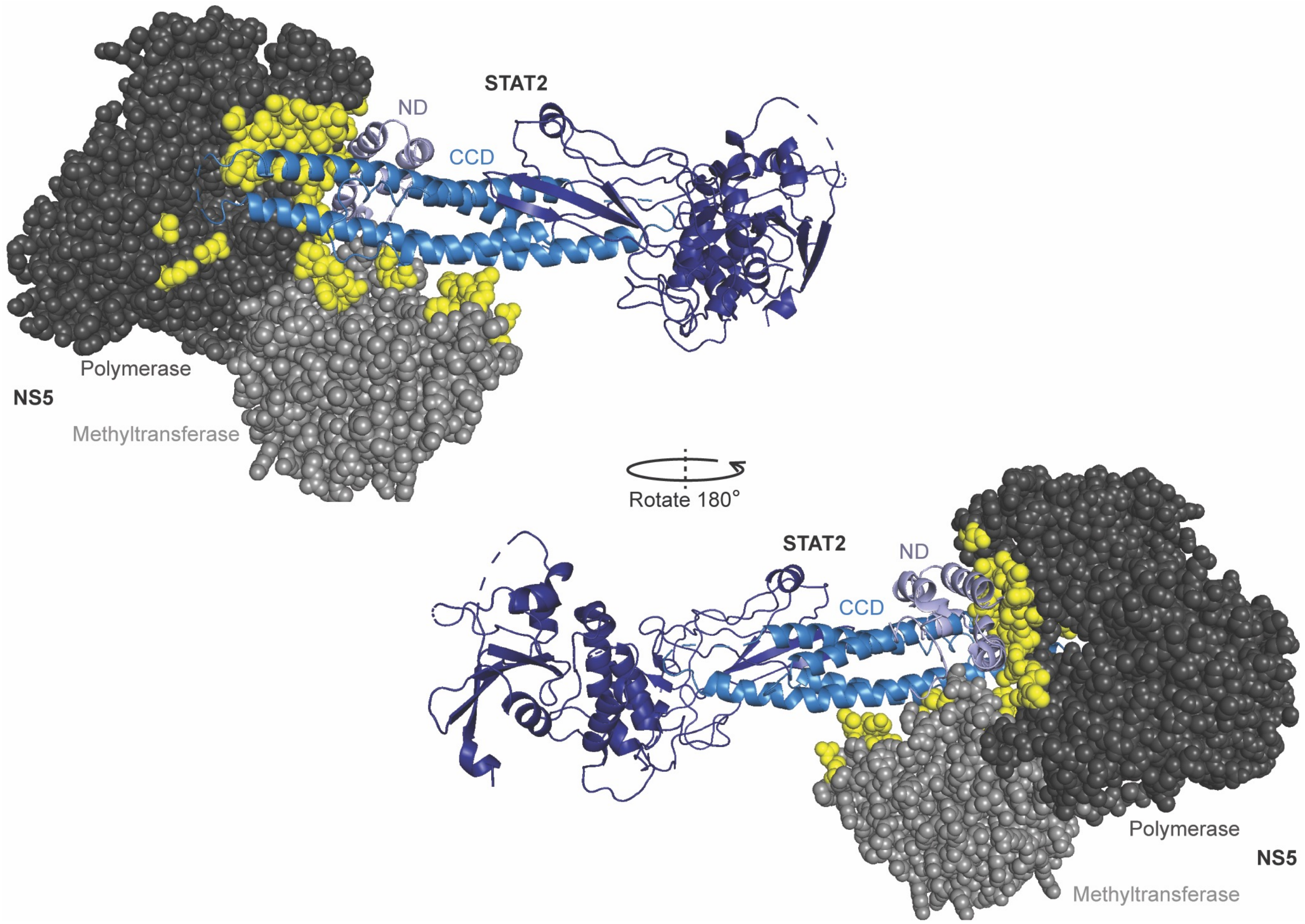

bioRxiv preprint doi: https://doi.org/10.64898/2025.12.23.696258; this version posted December 24, 2025. The copyright holder for this preprint (which was not certified by peer review) is the author/funder, who has granted bioRxiv a license to display the preprint in perpetuity. It is made available under aCC-BY 4.0 International license.

Supplementary Figure 2

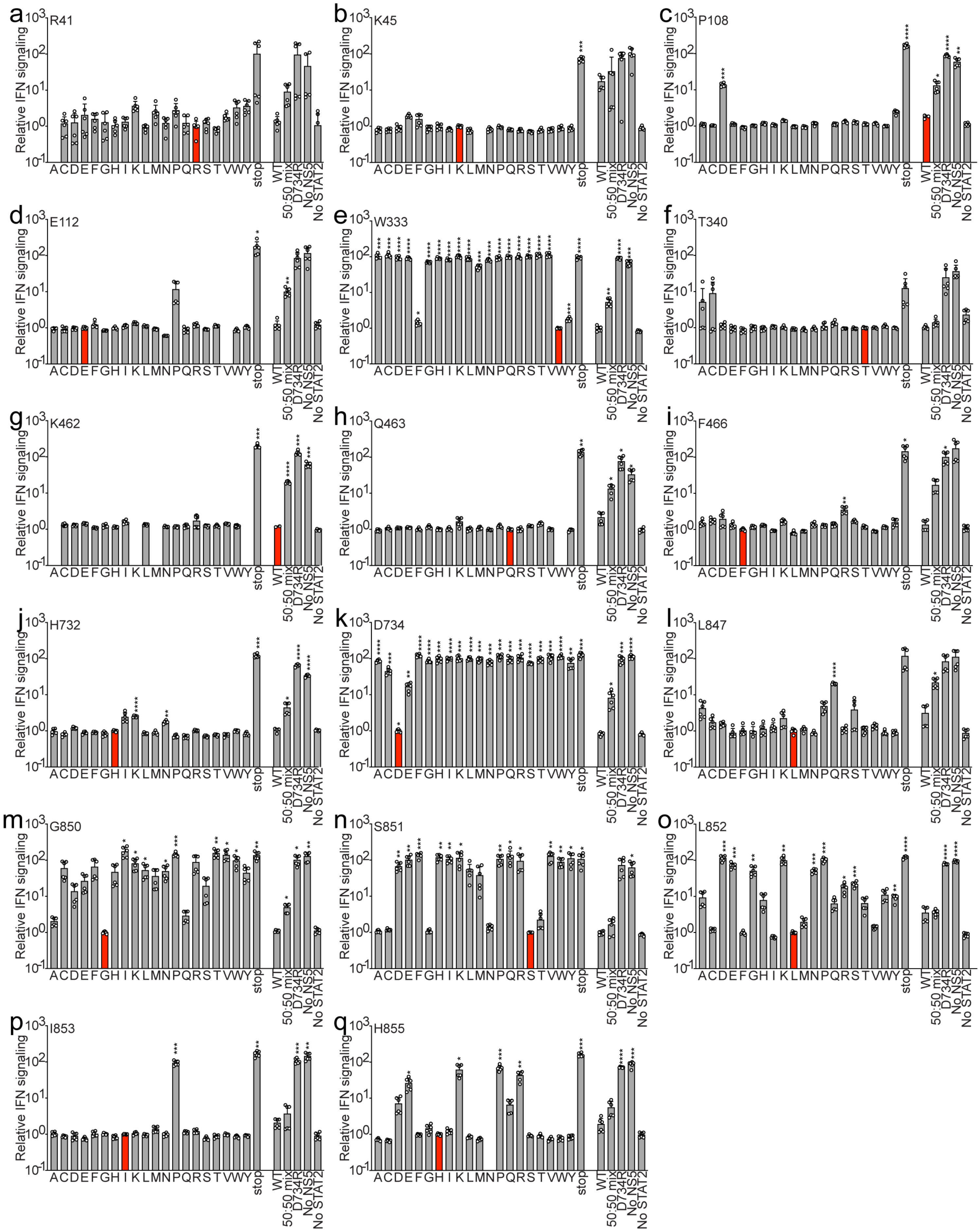

bioRxiv preprint doi: https://doi.org/10.64898/2025.12.23.696258; this version posted December 24, 2025. The copyright holder for this preprint (which was not certified by peer review) is the author/funder, who has granted bioRxiv a license to display the preprint in perpetuity. It is made available under aCC-BY 4.0 International license.

Supplementary Figure 3

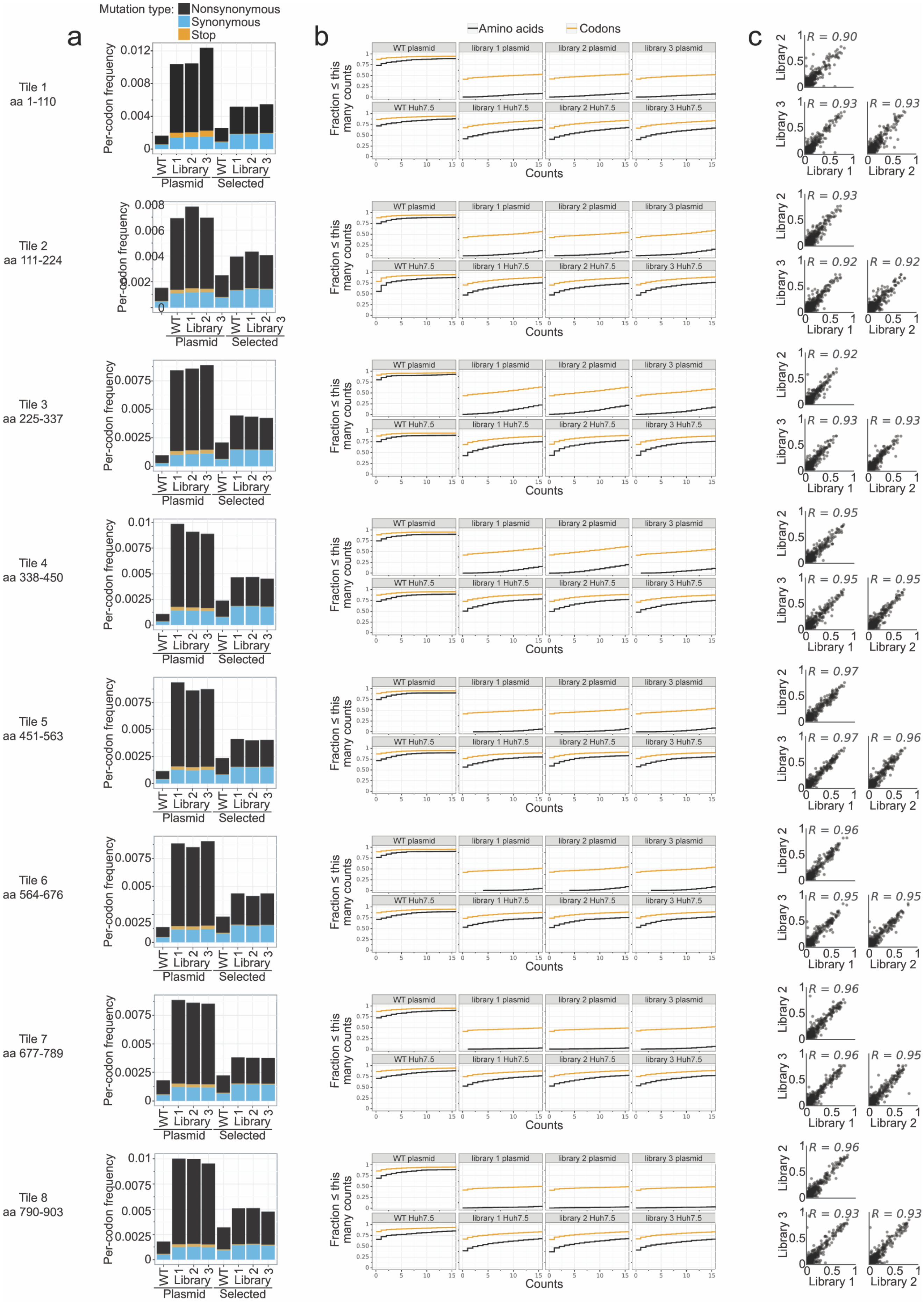

Supplement: Supplement 5 [file NIHPP2025.12.23.696258v1-supplement-5.pdf]
